# Impact of COVID-19 pandemic on sustainability determinants: A global trend

**DOI:** 10.1016/j.heliyon.2021.e05912

**Published:** 2021-01-09

**Authors:** Hafiz Syed Mohsin Abbas, Xiaodong Xu, Chunxia Sun, Atta Ullah, Samreen Gillani, Muhammad Ahsan Ali Raza

**Affiliations:** a College of Public Administration, Huazhong University of Science and Technology, Wuhan, 430074, Hubei, China; b School of Management, Huazhong University of Science and Technology, Wuhan, 430074, Hubei, China; c School of Economics, University of Central Punjab, Lahore, 54000, Pakistan; d School of Economics and Management, Beijing University of Posts and Telecommunications, Beijing, 100876, China

**Keywords:** COVID-19 pandemic, Sustainability, Vulnerable factors, Ageing population, People exposed to PM2.5% air pollution, Countries host the most international travelers

## Abstract

For the last six months till today, the world had had no luck in defeating COVID-19. This study examined the impact of the COVID-19 Pandemic on sustainability determinants, with the time arisen from December 27, 2019, through June 30, 2020. This study considers quantitative COVID-19 dashboard data with sustainable determinants; old age group, people exposed to air pollution, and countries with the most international travelers. Applying linear regression examines that COVID-19 behavior concerning the aging population and countries host the most international travelers, more positively significant than people exposed to PM2.5% air pollution, respectively**.** This study made a novel contribution by analyzing two variables' interaction; first, the aging population and the countries that host the most international travelers. Secondly, the aging population and people exposed to air pollution are vulnerable to COVID-19 globally, a novel concept comprehensively. Results show that countries with aging populations are more exposed to COVID-19, and its interaction term host the most international travelers. It also analyses that the aging population and its interaction with people exposed to air pollution are also vulnerable to COVID-19 but marginally lesser than the former. However, their behavior varies from country to country, making room for future study to analyze a more in-depth analysis. It gives a different dimension to consider other risk factors of COVID-19 by bearing in mind its unique contagious characteristics, which will help policymakers draft a sound epidemic preparedness policy to tackle the unforeseen crisis. It gives a thought of provoking to policy practitioners for the risk characteristics of COVID-19, which needs a reassessment to epidemic risk management to deal with this, and future unforeseen crisis by considering Sustainable Development Goals.

## Introduction

Sadly for the last six months until today, the world has been suffering from *COVID-19* (COVID-19). SARS-2 (Severe Acute Respiratory Syndrome) virus, which named nCov-19 or COVID-19 by World Health Organization. COVID-19. It first appeared in Wuhan City, China, on December 27, 2019, which gradually spread worldwide (The State Council of China, 2020). However, it remains a mystery for all scientists and experts where this virus originated. Furthermore, it has affected almost all age groups, genders, nationalities, and people of various income groups worldwide.

On January 21, 2020, by considering the frequency of COVID-19, WHO decided to issue a daily situation report of COVID-19 cases, which affected four countries. Finally, on January 30, 2020, WHO declared it a Global Health Emergency because more than 18 countries with 7,812 confirmed infected cases and 170 deaths worldwide had already reported. As COVID-19 had been uncontrollable and irresistible, on March 11, 2020, it was affirmed as Pandemic as it spread in more than 100 countries with 118 206 confirmed cases and 4,378 deaths, and it has been increasing day by day. As of June 30, 2020, 10,549,575 confirmed cases and 512,160 fatal cases, with a 55.1% recovered rate, had been reported worldwide.

WHO has been issuing information worldwide to raise awareness and guidelines for precautionary measures for public health and safety from time to time. WHO further issued guidelines and major vulnerable factors that expose to COVID-19; i.e., age group 60 years old and above, poor sanitation and unhygienic routine, people exposed to air pollution, people with flu and fever, and people who travel from infected areas. WHO also requested public figures to step forward and raise awareness among the public towards precautionary and safety measures against COVID-19.

COVID-19 Pandemic has been spreading with unstoppable and swift fashion in developed, high, and upper-middle-income group countries. However, countries with developed states have severely affected this virus because developed countries have not faced any such pandemic situation before. Although these developed American, European, and Western Asian countries have a better lifestyle, social status, and advance health response systems. However, the virus's unusual and strange behavior created a panic and chaos situation for governments worldwide. Gümüsay and Haack (2020) and Pan and Zhang (2020) suggest that this global Pandemic shares the uncertainty, vulnerability, and evaluability aspects that characterize major challenges and needs concerted and sustainable efforts different disciplines worldwide to resolve this global Pandemic. Kanda and Kivimaa (2020) recommended that from the sustainable environmental aspect, which effects are desirable in COVID-19 (e.g., reduced air travel) and what policies will help preserve the results? Such lessons will lead research into sustainability change by discussing the proliferation of emerging activities resulting from the sudden transformation of new practices, resilience, and relations between the global and the local economies. These and many more trajectories need to be discussed as we try to thoroughly tackle the COVID-19 Pandemic in the coming years and months.

This study examined the causation of COVID-19 regarding age group, environment exposure, and countries that most host international travelers. The study uses WHO vulnerable, or risk components, and the United Nations defined sustainable social determinants that could be affected the most under consideration for this study. COVID-19 is a novel SARS-2 that mostly affects the lungs and targets the immune system of the body. Moreover, Air pollution also affects the respiratory system of the human body during breathing when people visit gathering places. For this study, age group 60 and above, people exposed to air pollution and countries expose the most travelers have taken under consideration. This study examined the causality of the variables, as mentioned above, with the COVID-19 confirmed cases situation globally that has not studied the Pandemic's prevailing situation. Moreover, the interaction of aging pollution with international travelers and exposure to air pollution was also investigated in this study, making this study a novel contribution to COVID-19 literature and behavior.

The significance of the study is that COVID-19 has been showing indifference trends since its origination. This study aims to determine the relationship between real-time cases with age groups, the environment worldwide, and traveling causation with COVID-19. It will help policymakers and experts to review and focus on the possible susceptible components of COVID-19 specific age groups, environment regulations, and travel policies to combat future issues in various countries based on current trends. It will also help draw attention to sustainable development goals by taking appropriate government measures in the coming days, as studied by the scholars (Hale et al., 2020; Ashraf 2020). The paper structure is a research background, methodology, data and discussion, and conclusion with some policy implications on controlling such issues.

## Literature review

Hussain et al. (2020) have expressed concerns over COVID-19 by stating that it has rapidly spread since its initial identification in Wuhan. COVID-19 has shown a broad spectrum of severity; therefore, early isolation, early diagnosis, and early management might collectively contribute to better control of the disease and outcome. They suggest that future research is urgently needed better to understand potential differences in genetic predispositions across populations.

Different aged patients with COVID-19 infections with the lungs signs of identified cases compare in a recent study conducted in China and chest HRCT signs of infected patients in four age groups less than 18 years, between 18 to 44 years, between 45 to 59 years and greater than 60 years old compared. They find that the main HRCT signs in 98 patients with confirmed COVID-19 infections patients aged more than 60 years and between 45 to 59 years age group had more bilateral lung, field involvement in lungs, lung lobe, and lesion numbers than less than 18 years old patient. Also, in patients between 45 to 59 years and more than 60, the air Broncho gram sign was more common than in those aged less than 18.

Therefore conclude that HRCT signs are milder in younger patients and higher in older age patients (Chen et al., 2020a, Chen et al., 2020b; Vakili et al., 2020). Chen et al., 2020a, Chen et al., 2020b study findings show that children or young people have milder symptoms than older people, are easily cured and have a good prognosis than middle-aged and older people with COVID-19 infection. Li et al. (2020) survival analysis revealed that male and elder aged were associated with death in patients with severe COVID-19 and concluded that severe male patients might have a high risk of death with heart injury, hyperglycemia, and high-dose corticosteroid use. Lakhani (2020) findings suggest potential improvements in quality of care amid the Pandemic in Melbourne, Australia, to vulnerable populations, including elderly, palliative, and disabled patients, by utilizing GIS mapping to identify COVID-19 health care priority locations pertaining.

Recent studies across the world have shown that multiple factors may contribute to the severity and spreading rate of COVID- 19, such as Wu et al. (2020) showed that long-term air pollution exposure could exacerbate the health outcomes of COVID-19 cases. Wang et al. (2020) reported on environmental conditions by indicating that environmental conditions such as humidity, pollution, and temperature could influence the transmission of COVID-19 when compared to other respiratory viruses, suggesting a decline in disease spread in China. Overall reported findings of Wu et al. (2020), Wang et al. (2020), and Mollalo et al. (2020) suggest that air pollution exposure and pre-existing conditions may suffer from higher mortality risk. Xie and Zhu (2020) explored the relationship between ambient air pollutants and the infection caused by the novel coronavirus, and findings suggest that there is a significant relationship between air pollution and COVID-19 infection because air pollution is a risk factor for respiratory infection by carrying microorganisms and affecting body's immunity in China. Developing countries have a week management system posing potential COVID 19 spread (Adelodun et al., 2020).

Traveling around the world is suspended, which will undoubtedly create a decline in the travel index. The Global economies are also spiraling towards a recession in fear of this new life-threatening disease (de Alwis et al., 2020). Many distinct operational procedures and measures have been implemented concerning air traffic, while some countries have entirely shut down border crossings and restricted flights in affected areas. Some countries have implemented fever measurements and two-week-long self-quarantine for travelers to contain further spread of COVID-19 cases (Lau et al., 2020). Lau et al., 2020a, Lau et al., 2020b investigate this relation by analyzing available air traffic volume and the spread of COVID-19 cases. By comparing the distribution of domestic and international COVID-19 cases with current domestic and international passenger volume and flight routes, findings indicate a strong linear correlation between domestic COVID-19 cases and passenger volume for regions within China and a significant correlation between international COVID-19 cases and passenger volume. They suggest that we must critically re-evaluate air traffic restrictions and whether their continuous operation effectively increases global COVID-19 cases.

## Methodology

This study examined two demographic elements vulnerable to COVID-19 defined by the World Health Organization and under consideration to the United Nations in its sustainable development. This study has taken three dependent variables; the First is the age group aged 60 years old and above, and second, the people exposed to air pollution (Environment). Along with demographics, the third variable is a component of the Epidemic Risk Index (2020) that countries host the most international travelers. On the other hand, the study keenly analyzes the COVID-19 CDR (Confirmed, Deaths, and Recovered) cases (Explanatory variable) causation specifically with these three variables along with total population to case ratio analysis. Quantitative daily updated data for COVID-19 has been collected from December 27, 2019, through June 30, 2020, from the European Union Data portal, humanitarian data exchange, and John Hopkins University data updates supported by World Bank by considering WHO daily situations reports.

Data on total population and percentage of the population with age group 60 years old and people exposed to air population in percentage to the total population has extracted from the World Bank database (World Bank, 2020a & 2020b). For the countries exposed to travelers or which host the most international travelers as a component of epidemic risk index year 2020 issued by Inform risk management index has used in this study (INFORM 2020). This study analyzes the dashboard COVID-19 situation data with Risk factors of COVID-19 graphically in different manners.

This study consists of three analysis sections and is explained in detail. Moreover, in analysis, three dependent variables and one explanatory variable; first to analyze the worldwide causation of COVID-19 cases in 60 years old age group, second section deals with people exposed to air population and COVID-19 relationship and third, the countries with epidemic risk index in terms of exposure to international travelers and COVID-19 connection has analyzed. In this study, countries with more than one million population have selected to review the better demographic impact of COVID-19 on a massive scale and employed statistical analysis using STATA software and reported the descriptive statistics, Pearson's Correlation and Linear Regression.

## Data findings and discussion

### Graphical analysis

#### Section 1: COVID-19 trend and age group

**Figure 1:**
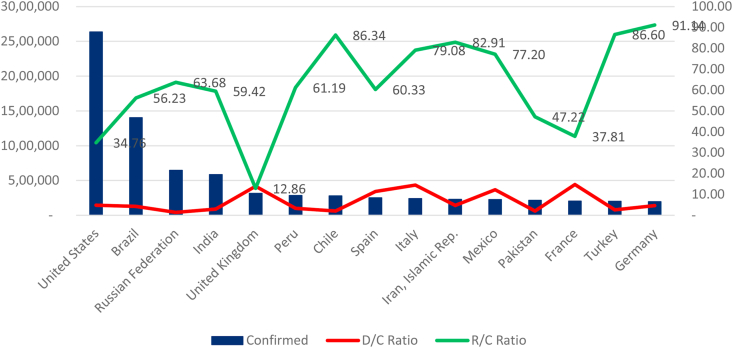
COVID-19 Situation analysis of the top 15 the most affected countries.

This section analyzes the real-time COVID-19 trend and countries with the most population with an age group 60 years and above as Figure 1 demonstrates the top 15 of the most affected countries' situation until June 30, 2020. The trending line depicts the recovered and fatal cases to the total confirmed cases ratio in the secondary y-axis. As per Statistics, the most affected overall countries are the USA, Brazil, Russia, India, and the U.K. while in terms of recovered cases China 94%, Germany 91%, Chile 86%, Turkey 86% and Iran 82%, performed better as compared to rest of the world. The U.K. situation is alarming in updated status, as the death rate is 14% higher than the recovered rate of 12%. Furthermore, the U.K. has the highest fatality rate and lower recovered rate globally, which raises doubts about the health system and immunity of the British people.

However, deepen contrasting depictions of confirmed cases to population ratio analysis revealed that where the world is focusing on the American and European states, some other small European and Western Asian states observed the most vulnerable in this contrast. Qatar and Bahrain are the most vulnerable in population to confirmed case ratio depiction. In this contrast, the United States ranked in the ninth place where every 124^th^ person is affected with COVID-19. Apart from the most affected countries, this population to cases ratio analysis, Qatar, Bahrain, Chile, Kuwait, and Peru emerged the most vulnerable countries with every 29^th^, 59^th^, 67^th^, 90^th,^ and 112^th^ person is affected in the top 15 countries in population to confirmed cases ratio analysis. In the opposite scenario, mostly Asian countries show a high population to case ratio even in China, where every 16,427^th^ person is affected. However, in the overall situation analysis, pictures are different.

**Figure 2:**
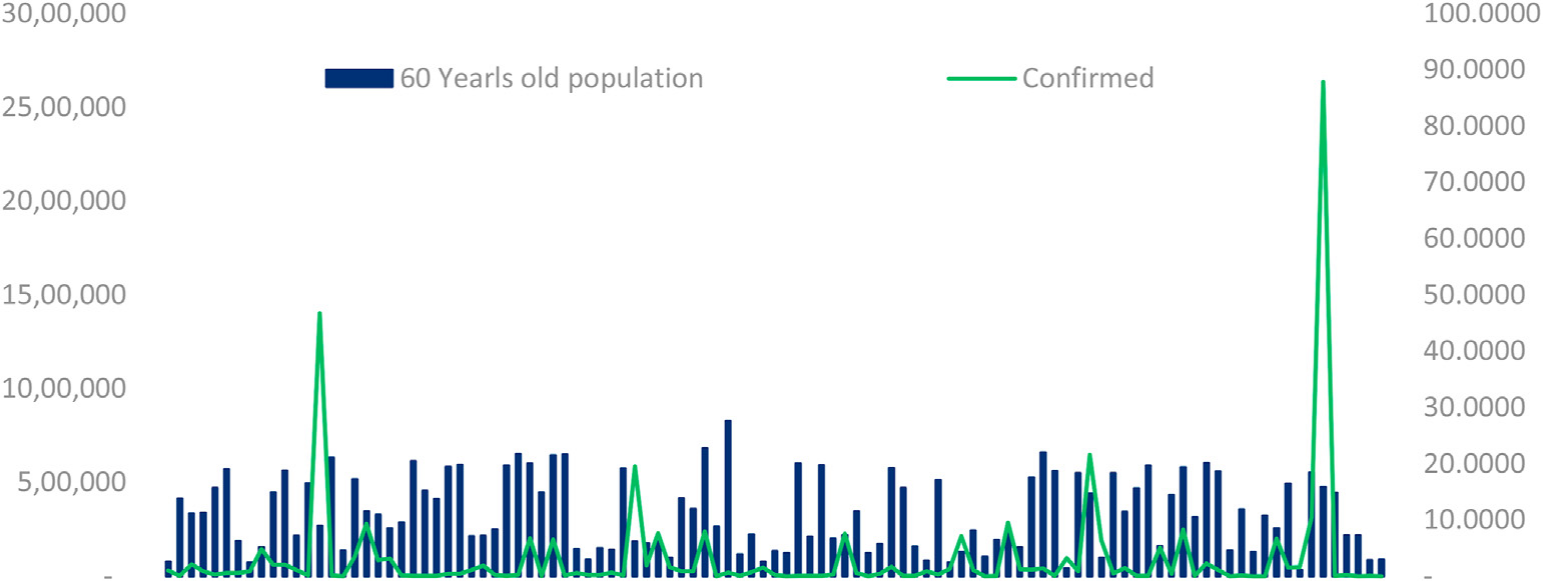
Global Analysis of 60 years old age population to confirmed cases, Authors estimation.

Another demographic analysis explains the WHO statement of the most vulnerable age group population in Figure 2. This analysis shows that countries having the most number of inhabitants with the age group 60 years old and confirmed cases. As per this contrast, Italy, Germany, and France are the only countries that fulfill WHO's criteria of the vulnerability of age bracket in the most affected countries with 60 years old population. While this 60 years old contrasting analysis can be seen with a different perspective to analyze this causation, it further analyzed that the most affected countries with 60 years old population to confirmed cases ratio. Italy, Germany, Spain, France, and the Netherlands are the most affected countries with 60 years old population. Summing up the demographic situation estimation of COVID-19 revealed that real-time situation trends are somehow different from WHO's vulnerability group prediction towards COVID-19 Pandemic spreading and contagious characteristics. However, European countries and western Asian countries are the most vulnerable countries in demographic to confirmed cases contrasting analysis.

#### Section 2: COVID-19 analysis with PM2.5 concentration or countries with more exposure to air pollution

**Figure 3:**
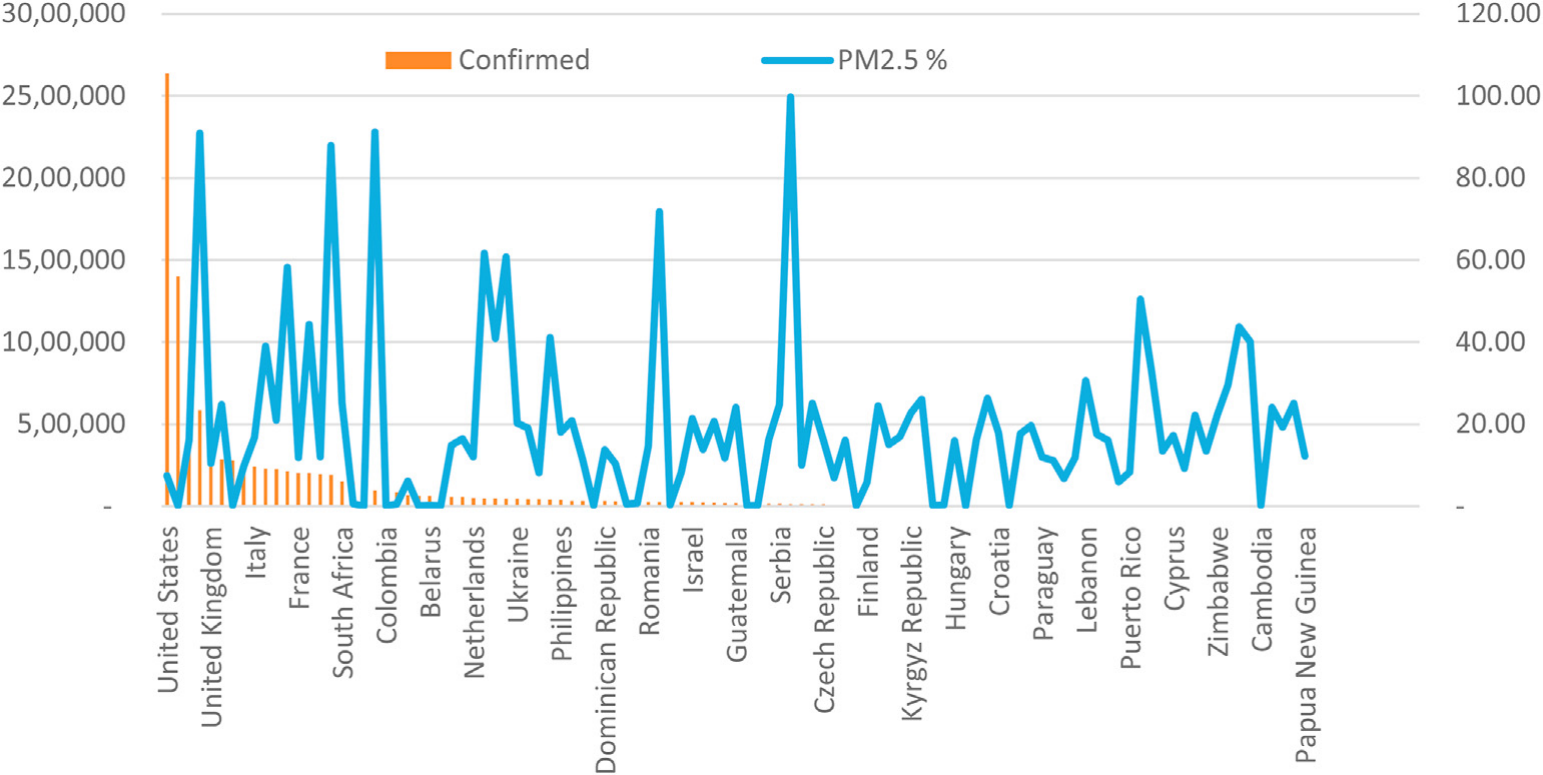
Global Analysis Air pollution and COVID-19 Situation, Authors Estimation.

This section explains the countries with the most exposure to the air population (PM2.5 concentration) in Figure 3. However, as per statistics, none of the most affected countries of COVID-19 is on the list of most air polluted countries except India, Turkey, and Iran. As per the WHO's estimation, the most exposed to air pollution people are in the vulnerable group to COVID-19. It further explained that the Countries with exposure to air pollution to confirmed cases ratio. The analysis showed that the least air polluted countries with PM2.5 concentration are the most affected countries list, which is not somehow in favor of vulnerability estimation of WHO regarding COVID-19 Pandemic. These statistics showed that countries with low air pollution had been severely affected by a global perspective compared to highly polluted countries except a few of them.

#### Section 3 described the COVID-19 and countries with most host the most international travelers via air traffic

**Figure 4:**
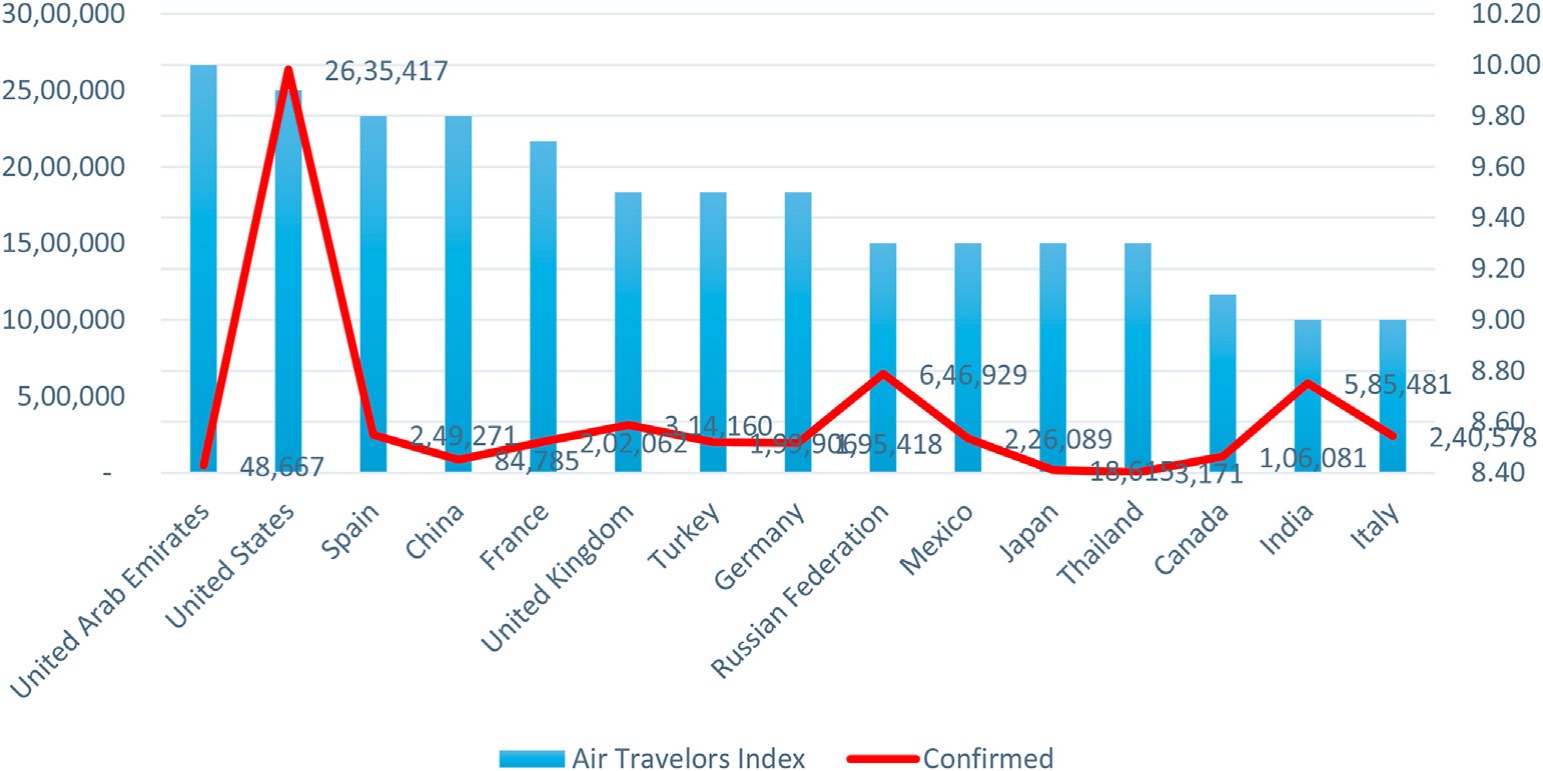
Top 15 the most host the international travelers and COVID-19 Situation, Authors Estimation.

**Figure 5:**
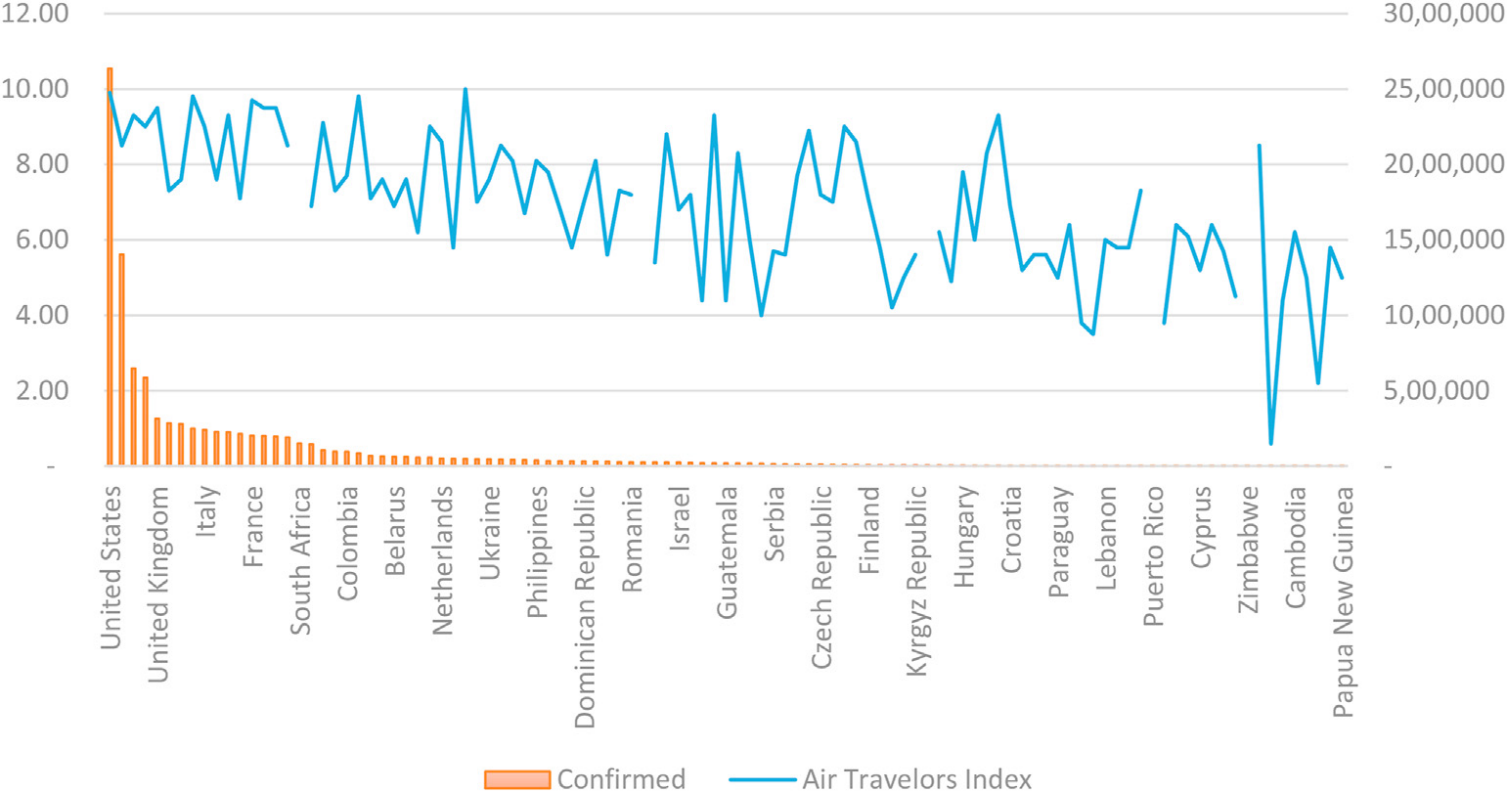
Global Analysis of COVID-19 situation and Air travelers' index.

**Figure 6:**
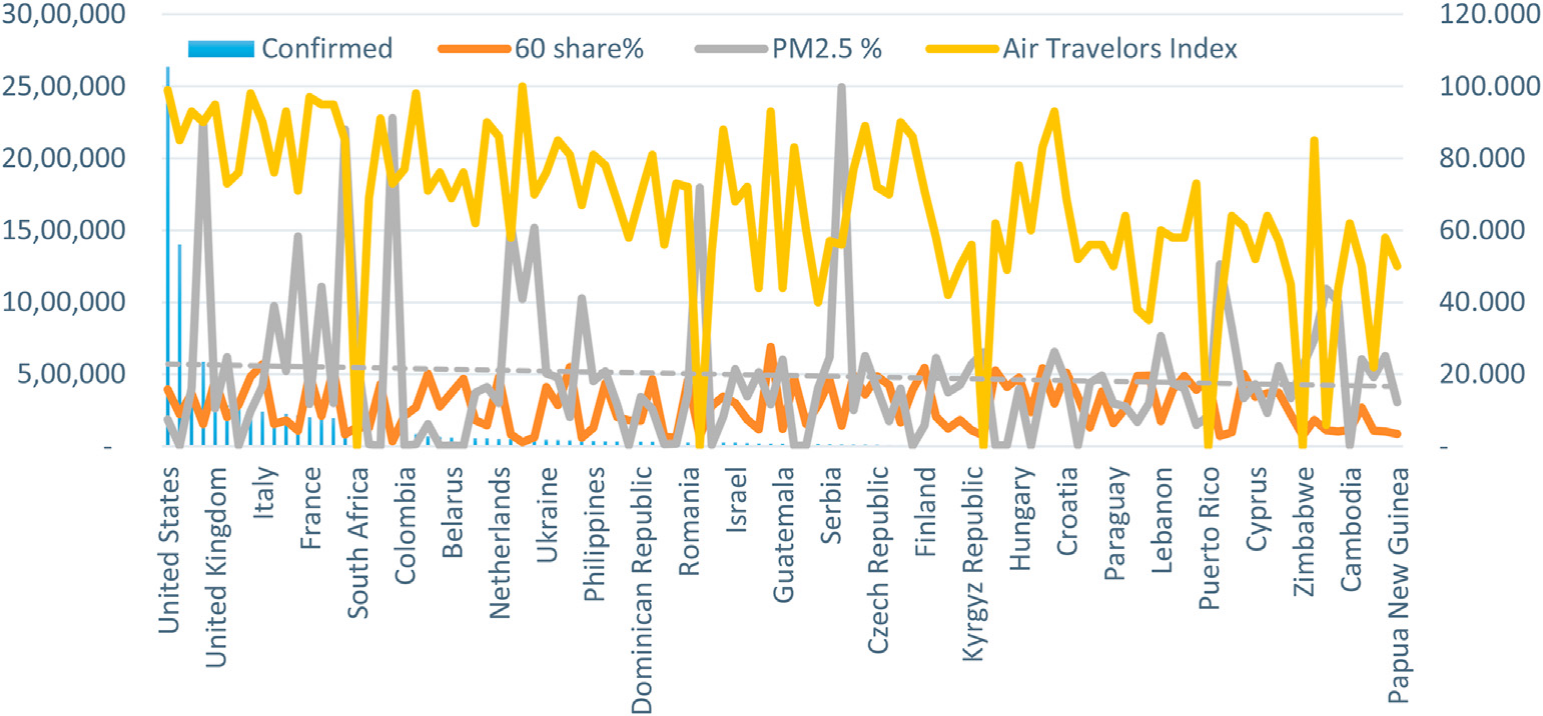
A global Comparative Analysis of Risk factors and COVID-19 Situation, Authors Estimation.

Figure 4 showed an epidemic index variable, which is likely to become the cause of COVID-19 widespread; as per WHO situational reports and analysis, most countries were affected due to international travelers or imported transmitted cases. Statistics revealed that the UAE host the most international travelers; however, its COVID-19 infection is not at the higher side so far, but troublesome. On the other hand, most countries with a high air traveler vulnerability index have been severely infected by COVID-19. It examined that 9 out of 15 countries with high air traveler's vulnerability index are in the top list of affected countries with COVID-19, supporting the Inform risk management index of the epidemic index. The U.S. and other European countries such as Spain, Italy, Germany, France, Russia, U.K., and Turkey are top in the most affected countries from COVID-19 so far and host the most international travelers around the globe. Figure 5 showed the global situation analysis of COVID-19 and Air Travelers Index (see Figure 6).

By summed up all this commentary, as per the recent trend of COVID-19 pandemic situation to epidemic risk assessment factor of the host, the air travelers showed much significant 0.4216 and impacted by COVID-19 Pandemic spreading much higher the other risk factors. That is why most countries at the early stage of precautionary measures announced flight operations' closure for international travelers. In recent of Kanda and Kivimaa (2020) conclude that the crisis may encourage the systems of governance to be better equipped for various sorts of disruptions like the socio-technical system in the long run, while it also threatens largely on policies stability and increased securitization. The current study of almost every country banned international travelers due to risk factors and increased the traffic security system.

### Statistical analysis

Based on discussion and paper concept. Table 1 described the descriptive statistics of the log values of the data.

**Table 1:** Descriptive statistics. Authors estimation.

Variables	Obs	Mean	Std. Dev.	Min	Max
Year	105	2020	0	2020	2020
Log-COVID-19 Confirmed Cases	105	9.457	2.361	2.398	14.784
Log-Population 60 above	105	14.152	1.607	10.547	18.840
Log-Population Exposed to Air Pollution	105	14.390	1.814	10.644	20.413
Log-Air Traveler Index (ATI)	102	1.880	0.364	0.510	2.302
Log-Population 60 above∗ATI	102	26.363	7.15	6.000	43.000
Log-Population 60 above∗Pollution	105	205.229	46.965	127.000	384.000

This study has used three dependent risk factors of COVID-19 and one independent variable. A simplified Pearson Correlation and Linear Pool regression Analysis has been investigated. Table 2 explained the Correlation results and observed that COVID-19 infection cases have positively correlated with the countries having a population over 60 years old, population exposed to PM2.5% Air pollution, and host air travelers the most.

**Table 2:** Pearson correlation analysis: Source: Authors estimation.

	Log-COVID-19 Confirmed Cases	Log-Population over 60	Log-Population exposed to Air Pollution	Log-Air Traveler Index (ATI)
Log-COVID-19 Confirmed Cases	1.000			
Log-Population over 60	0.587	1.000		
Log-Population exposed to Air Pollution	0.537	0.692	1.000	
Log-Air Traveler Index (ATI)	0.566	0.511	0.323	1.000

#### Linear Pool regression

In a more profound analysis, a Linear Regression has been made in Tables 3, 4, and 5. The description of the tests in three sections is graphically explained earlier. The first section, Impact of Confirmed COVID-19 Cases on Population over 60 years old to total population ratio, shows the R-square Value 0.3449 with a 95% confidence interval. In section two: Impact of Confirmed COVID-19 Cases on Population Exposed to PM2.5% Pollution with 0.2888 R-square value with 95% confidence interval and in third section Impact of Confirmed COVID-19 Cases on Air Travelers Index and observed an R-square value 0.3204 with 95% confidence interval. The regression analysis concludes that countries with an aging population are more affected in the COVID-19 Pandemic and host the most air travelers in their countries than polluted countries.

**Table 3:** Linear regression of population over 60 Years old. Source: Authors estimation.

Source	SS	DF	MS	Number of Obs	105
Model	92.575	1	92.575	F (1,103)	54.22
				Prob > F	0
Residual	175.861	103	1.707	R-Squared	0.344
				Adj R-Squared	0.339
Total	268.437	104	2.581	Root MSE	1.306

Log-Population over 60	Coef.	Std.Err.	t	p > t	[95% conf. Interval]	
Log-COVID-19 Confirmed Cases	0.400	0.542	7.360	0.000	0.291	0.507
Constant	10.373	0.529	19.620	0.000	9.325	11.422

**Table 4:** Linear regression of population exposed to pollution. Source: Authors estimation.

Source	SS	DF	MS	Number of Obs	105
Model	98.888	1	98.888	F (1,103)	41.82
				Prob > F	0.000
Residual	243.554	103	2.364	R-Squared	0.288
				Adj R-Squared	0.281
Total	342.442	104	3.292	Root MSE	1.537

Log-Population exposed to Air Pollution	Coef.	Std.Err.	t	p > t	[95%conf. Interval]	
Log-COVID-19 Confirmed Cases	0.412	0.063	6.47	0.000	0.286	0.539
Constant	10.484	0.622	16.85	0.000	9.25	11.719

**Table 5:** Linear regression of air traveler index. Source: Authors estimation.

Source	SS	DF	MS	Number of Obs	102
Model	4.292	1	4.292	F (1,100)	47.150
				Prob > F	0.000
Residual	9.103	100	0.91	R-Squared	0.320
				Adj R-Squared	0.313
Total	13.4	101	0.132	Root MSE	0.301

Log-Air Traveler Index	Coef.	Std.Err.	t	p > t	[95%conf. Interval]	
Log-COVID-19 Confirmed Cases	0.087	0.012	6.87	0.000	0.062	0.112
Constant	1.054	0.123	8.52	0.000	0.808	1.3

#### Linear pool regression analysis with integration terms

By analyzing the vulnerable factors behaving worldwide, this study investigated two integration terms and observed the COVID-19 impact on them. First: Population over 60 years old and countries host the most international travelers. It is observed that COVID-19 most infects countries with having an aging population and host international travelers with R-square value 0.4174 in Table 6. Second: Population over 60 years old and exposed to air Pollution the most, in this scenario as compared to Tables 3 and 4, aging population exposed to air pollution are more vulnerable to COVID-19 with R-square value 0.3551 in Table 7.

**Table 6:** Log-Population over 60 years∗Log-Air Travelers Index. Source: Authors Estimation.

Source	SS	DF	MS	Number of Obs	102
Model	2155.341	1	2155.341	F (1,100)	71.650
				Prob > F	0.000
Residual	3008.237	100	30.082	R-Squared	0.417
				Adj R-Squared	0.411
Total	5163.578	101	51.124	Root MSE	5.484

Log-Population over 60∗Air Traveler Index	Coef.	Std.Err.	t	p > t	[95%conf. Interval]	
Log-COVID-19 Confirmed Cases	1.955	0.230	8.460	0.000	1.497	2.413
Constant	7.875	2.250	3.500	0.000	3.410	12.340

**Table 7:** Log-Population over 60 years old∗Log-Pollution Expose to Air Pollution. Source: Authors Estimation.

Source	SS	DF	MS	Number of Obs	105
Model	81457.856	1	81457.856	F (1,100)	56.710
				Prob > F	0.000
Residual	147940.658	103	1436.317	R-Squared	0.355
				Adj R-Squared	0.349
Total	229398.514	104	2205.754	Root MSE	37.899

Log-Population over 60∗population exposed to Air Pollution	Coef.	Std.Err.	t	p > t	[95%conf. Interval]	
Log-COVID-19 Confirmed Cases	11.852	1.573	7.530	0.000	8.730	14.973
Constant	93.147	15.335	6.070	0.000	62.732	123.561

### Discussion

It analyzed that COVID-19 has swiftly concerted into Pandemic from an endemic. Initially, scientists and experts estimated that countries with bad health and sanitation system would be most susceptible to this virus. However, its distinguished dissemination fashion made the global scientists and experts worrisome. It analyzed that countries with developed political and national systems are poorly affected, and their health and preparedness policies failed to tackle this Pandemic. People in the old age group are estimated to be vulnerable to this virus (Hussain et al., 2020; Lakhani, 2020). The disorder's general vulnerability, the clinical characteristics, and laboratory findings on various age groups are different but more in older people. Besides that (Richardson et al., 2020; Wu and McGoogan 2020) concluded that a striking phenomenon in COVID-19 was that people aged 60 or older suffered the most onerous disease burden. Moreover, American and European states are poorly affected due to this Pandemic; however, as per population to confirmed cases, ratios describe different situations. It examined that some European and Western Asian countries have a worst situation than other developing and underdeveloped countries. It is an unusual or unpredictable drift with little support from the WHO's initial statement towards COVID-19 contagion and transmission.

Analysis outcomes showed the causation of air pollution and COVID-19. It is a general assumption that countries with high pollution and PM2.5 concentration in the air are vulnerable to respiratory diseases and other lung-related issues (Lakhani, 2020; Wu et al., 2020). However, the recent COVID-19 trend mostly suppressed this assumption and showed somehow different trends. Results show that countries with the least air pollution or people with the least exposure to air pollution are the most affected due to COVID-19 compared to the aging trend. Moreover, people with the most exposure to air pollution are mostly least affected by this virus in recent times in the initial six months of COVID-19 Pandemic. The third variable, countries, are hosting the most international air travelers, which is the probable spreading factor of this Pandemic, somehow counterpart to COVID-19 trend (Lau et al., 2020). The major European countries, the USA, India, and China, are in the top list and have been affected the most from COVID-19, except the UAE in the first rank. While its susceptibility towards the virus is the lessor as compared to other American, European and Asian countries, even other Middle East countries, however, it also emerged another dimension of study COVID-19 that are these most affected countries became the carrier of this virus to other small or developing countries due to their epidemic index scale?

As per the graphical and regression analysis, this study has observed the following trends. A global graphical analysis showed that countries with the host the most are affected the most from COVID-19 while in regression analysis, its vulnerable factors are the second most determinant of COVID-19 after the aging population. However, their interaction in Table 6 demonstrated that countries with an aging population and host the most international travelers exposed the most to COVID-19. At the same time, countries exposed to air pollution are less affected from COVID-19 in the three selected factors as depicted in Figures 3 and 4. However, their interaction with the aging population, as mentioned in Table 7, enhances the vulnerability to expose COVID-19.

However, this study made an unusual analysis of the aging population's interaction with air travelers and air pollution. These findings show that countries with an aging population and host the most international travelers in its initial six months are most affected by COVID-19 while the aging population by exposure to air pollution is less affected than the former. However, it empirically concludes that countries with aging populations are more infected by COVID-19, and other social interaction determinants have been enhancing its susceptibility (Lanese 2020).

## Conclusion and policy implications

This study examined the causation of COVID-19 and sustainability determinants - age bracket 60 years and old, population exposed to pollution, and countries host the most International travelers) in the global perspective. By reviewing the crucial aspect of COVID behavior, it found the indifference trend of WHO and the U.N.'s estimation towards COVID-19. This study has contributed to the existing COVID-19 vulnerability investigation by taking two interaction analyses of the aging population with air travelers and air pollution globally. The COVID-19 is still devastating global economies and health systems, which need a more in-depth global analysis to study the vulnerable factors which have not been studied yet or give little attention. Hence, comprehensive risk factors analysis is a dire need to stop the coming COVID-19 waves and other unforeseen future calamities.

This study revealed an essential aspect of a COVID-19 pandemic that is ignored or unattended yet. The world was focusing on the USA and other European states with their total numbers of affected cases and did not discuss the demographic concentration and age bracket ratio. Middle-East and some least prominent European states should deal with equal global supports, as the population to confirmed case ratios and those where the population with 60 years old to confirmed case ratios are going fewer day by day will be most dangerous in the coming days. The same pattern of distinct features observed in the least air polluted countries which are affected the most.

This study concluded with two findings; the first initial WHO estimation towards COVID-19 patterns was not good enough or was too early to predict this virus's behavior or yet some other factors are most vulnerable with COVID-19 Pandemic spreading. Secondly, most likely, this virus's unique characteristic. As per the WHO and other global scientists, this novel coronas virus has some unique contagious characteristics that had not been studied or observed before, making it difficult to control or examine its actual pattern. That is why current globalized modern medical science is unable to find out its cure yet. The only known possibility of its spreading discovered yet is its contagious nature, which can be controlled via social distancing and staying at home. However, this strategy is workable in developed and prosperous countries and fails in struggling countries due to their socioeconomic crisis; currently, the WHO formed a team of global scientists and researchers to develop the vaccine.

The previous studies examined that COVID-19's changing environment could lead to greater localization and greater sustainability. For mobility, the long-term consequences of the Pandemic on sustainability can be linked to more permanent changes related to the digitization of work and other daily activities, which reduce mobility requirements such as air travel in particular. Also, the current study endorses the critical remarks of the latest studies by emphasizing that in light of COVID-19 substantial damage to human life and livelihoods, the coronavirus pandemic gives the human family an excellent opportunity to act in unison and make this Pandemic the guiding force for the United Nations (U.N.) Sustainable Development Goals (SDG). We hope that global governing and recommended agencies and resource-rich countries will discover or develop its cure or vaccine soon. Through this good global cooperation, this Pandemic will be controlled or eradicated.

Nevertheless, COVID-19 has been changing so rapidly, and governments worldwide have been trying their best to control it. In this regard, along with social distancing as prescribed by the WHO, an internationally shared governance concept is needed. The private companies should bring their efforts and support to the government to overcome this Pandemic. In China, along with Public-Private Partnership, substantial private support, e.g., Alibaba and Tencent group, gave their services and support to the government and general public by sharing the state's socioeconomic burden. Moreover, aggressive public testing at government expense or minimum cost mitigates the effects of COVID-19.

### Future study and limitation

This study used only three sustainable factors to analyze more sustainable development factors that can be taken for a more comprehensive examination with COVID-19. This study has some limitations, as this study made a global analysis that cannot cater to country government response. However, a regional analysis or impact of certain policies on combating COVDI-19 can be analyzed in future studies for single or small samples. Further, this study examined the confirmed cases or affected countries' situation with a demographic, environmental, and traveling status worldwide. For the future study direction, this demographic or other different characteristics and deaths and recovery trends or patterns can be studied globally or in individual countries regarding the COVID-19 Pandemic. Moreover, government responses with COVID-19 situation analysis will also be an excellent dimension to analyze COVID-19 behavior. In this regard, the Oxford University team's recently developed government response and policy index can be used.

## Declarations

### Author contribution statement

Hafiz Syed Mohsin Abbas: Conceived and designed the experiments; Wrote the paper.

Xiaodong Xu, Muhammad Ahsan Ali Raza: Analyzed and interpreted the data.

Chunxia Sun, Atta Ullah: Contributed reagents, materials, analysis tools or data.

Samreen Gillani: Performed the experiments; Analyzed and interpreted the data.

### Funding statement

This work was supported by: Research on Intelligent Mode of Social Governance in City Area (20AZD089), National Social Science Foundation Key Project. Grant no.: 20AZD089. Recipient of Grant: Prof. Dr. Xiaodong Xu.

### Data availability statement

Data will be made available on request.

### Declaration of interests statement

The authors declare no conflict of interest.

### Additional information

No additional information is available for this paper.
